# Drought tolerance of the grapevine, *Vitis champinii* cv. Ramsey, is associated with higher photosynthesis and greater transcriptomic responsiveness of abscisic acid biosynthesis and signaling

**DOI:** 10.1186/s12870-019-2012-7

**Published:** 2020-02-04

**Authors:** Noé Cochetel, Ryan Ghan, Haley S. Toups, Asfaw Degu, Richard L. Tillett, Karen A. Schlauch, Grant R. Cramer

**Affiliations:** 1 Department of Biochemistry and Molecular Biology, University of Nevada, Reno, NV 89557 USA; 2 Present address: College of Agriculture and Environmental Sciences, Bahir Dar University, Bahir Dar, Ethiopia

**Keywords:** Abscisic acid, Drought, Galactinol synthases, Grapevine, Transcriptomics, *Vitis*, Water deficit

## Abstract

**Background:**

Grapevine is an economically important crop for which yield and berry quality is strongly affected by climate change. Large variations in drought tolerance exist across *Vitis* species. Some of these species are used as rootstock to enhance abiotic and biotic stress tolerance. In this study, we investigated the physiological and transcriptomic responses to water deficit of four different genotypes that differ in drought tolerance: Ramsey (*Vitis champinii*), Riparia Gloire (*Vitis riparia*), Cabernet Sauvignon (*Vitis vinifera*), and SC2 (*Vitis vinifera x Vitis girdiana*).

**Results:**

Ramsey was particularly more drought tolerant than the other three genotypes. Ramsey maintained a higher stomatal conductance and photosynthesis at equivalent levels of moderate water deficit. We identified specific and common transcriptomic responses shared among the four different *Vitis* species using RNA sequencing analysis. A weighted gene co-expression analysis identified a water deficit core gene set with the ABA biosynthesis and signaling genes, *NCED3*, *RD29B* and *ABI1* as potential hub genes. The transcript abundance of many abscisic acid metabolism and signaling genes was strongly increased by water deficit along with genes associated with lipid metabolism, galactinol synthases and MIP family proteins. This response occurred at smaller water deficits in Ramsey and with higher transcript abundance than the other genotypes. A number of aquaporin genes displayed differential and unique responses to water deficit in Ramsey leaves. Genes involved in cysteine biosynthesis and metabolism were constitutively higher in the roots of Ramsey; thus, linking the gene expression of a known factor that influences ABA biosynthesis to this genotype’s increased *NCED3* transcript abundance.

**Conclusion:**

The drought tolerant Ramsey maintained higher photosynthesis at equivalent water deficit than the three other grapevine genotypes. Ramsey was more responsive to water deficit; its transcriptome responded at smaller water deficits, whereas the other genotypes did not respond until more severe water deficits were reached. There was a common core gene network responding to water deficit for all genotypes that included ABA metabolism and signaling. The gene clusters and sub-networks identified in this work represent interesting gene lists to explore and to better understand drought tolerance molecular mechanisms.

**Electronic supplementary material:**

The online version of this article (10.1186/s12870-019-2012-7) contains supplementary material, which is available to authorized users.

## Background

Water deficit (WD) has negative impacts on crop growth and yield [1] and affects crop quality traits [2]. Plants evolved adaptive mechanisms to cope with water scarcity. They can (i) escape the WD with a short crop cycle, (ii) avoid the WD (reducing transpiration/increasing water uptake), (iii) maintain growth or (iv) resist severe WD conditions by survival mechanisms [3]. In viticulture, seasonal drought, combining atmospheric and edaphic constraints, have large impacts on yield, grape berries’ organoleptic properties and subsequent wine quality [4]. Grapevine is mostly considered as a drought avoiding species [4, 5]. However, a large variability exists among *Vitis* species regarding this adaptive strategy ranging from isohydric (pessimistic), controlling water loss when soil water content decreases to anisohydric (optimistic), maintaining stomatal aperture and photosynthesis for the same WD conditions leading to a drop in leaf water potential [5, 6]. This classification is not strict and largely relies on the duration and intensity of the WD along with the environmental conditions [7]. In vineyards, drought tolerance can be ameliorated by using rootstocks [8]. Some of these *Vitis* species enhance the growth of *Vitis vinifera* scion cultivars in semi-dry and dry climates even with low irrigation [4]. Grapevine serves as a good plant model to study drought tolerance because of the large genetic variability that exists amongst these species and it is a valuable fruit crop [9].

Stomatal control in grapevine depends on hydraulic and chemical signals [10]. However, a comprehensive model depicting the relative importance of both processes remains to be investigated. Tombesi et al. [11] suggested that the immediate response relies on hydraulic mechanisms while the long-term regulation of transpiration involves abscisic acid (ABA). Rossdeutsch et al. [12] further confirmed the importance of ABA and highlighted a genetic background separation of grapevine genotypes for ABA-mediated responses in WD conditions [12]. Recently defined drought-responsive gene subnetworks identify the preponderant role of ABA signaling actors [13]. A recent report showed that the ABA action on grape leaf hydraulic conductance depends on the (an) isohydric behavior of the plant [14]. This further highlights the genetic background influence on the ABA-mediated grapevine response to WD.

The ABA metabolism and signaling pathways are well characterized. ABA belongs to the group of metabolites called terpenoids and its biosynthesis mostly relies on the activity of nine-cis-epoxycarotenoid dioxygenase (NCED) enzymes [15–17]. In addition to its production, catabolism is an important process for ABA homeostasis. ABA can be stored in an inactive form as ABA-glucose ester (ABA-GE) and re-activated rapidly through glucosidase activity [18]. ABA degradation is triggered by ABA-8′-hydroxylases [17]. Interestingly, ABA catabolites were suggested to be involved in ABA long-term responses [19]. The core signaling pathway is well described [20–22]. ABA signaling involves a complex regulatory network including a plethora of proteins [23].

Depending on their genetic background, the regulation of the ABA pathways could be different among *Vitis* genotypes and it could contribute to their differences in drought tolerance. However, ABA is not the only factor affecting plant response to WD. Indeed, the ability to perceive and transmit the drought stress related signal to the shoot part is also crucial and could rely on several other key players such as hydraulic signals, pH, ions, small peptides and other hormones [23–28]. To investigate the physiological and the molecular mechanisms differing between drought sensitive and drought tolerant grapevines, four genotypes were selected: Riparia Gloire (*Vitis riparia*; RG), known to be drought sensitive [29], Cabernet Sauvignon (*Vitis vinifera*; CS) considered an intermediate and Ramsey (*Vitis chamipinii*; RM), described as drought tolerant [30, 31]. The last genotype, SC2 (SC), is native to Southern Nevada; it is a hybrid of *Vitis vinifera* and *Vitis girdiana* discovered in the desert by Dr. Andrew Walker (personal communication). WD and recovery experiments were conducted on vines with different ages and different growth conditions. Physiological measurements were coupled with a transcriptomic study to gain insights into the molecular actors involved in the physiological processes that respond to WD conditions in the four different selected *Vitis* species. The main goals were (*i*) to identify the physiological response to WD of different *Vitis* genotypes ranging from drought-sensitive to drought-tolerant, and (*ii*) to correlate the transcriptomic response of these four species with drought tolerance to gain a deeper understanding of the physiological and molecular mechanisms involved.

## Results

### Photosynthesis was higher and maintained longer in RM with moderate WD in big pots

**Fig. 1:**
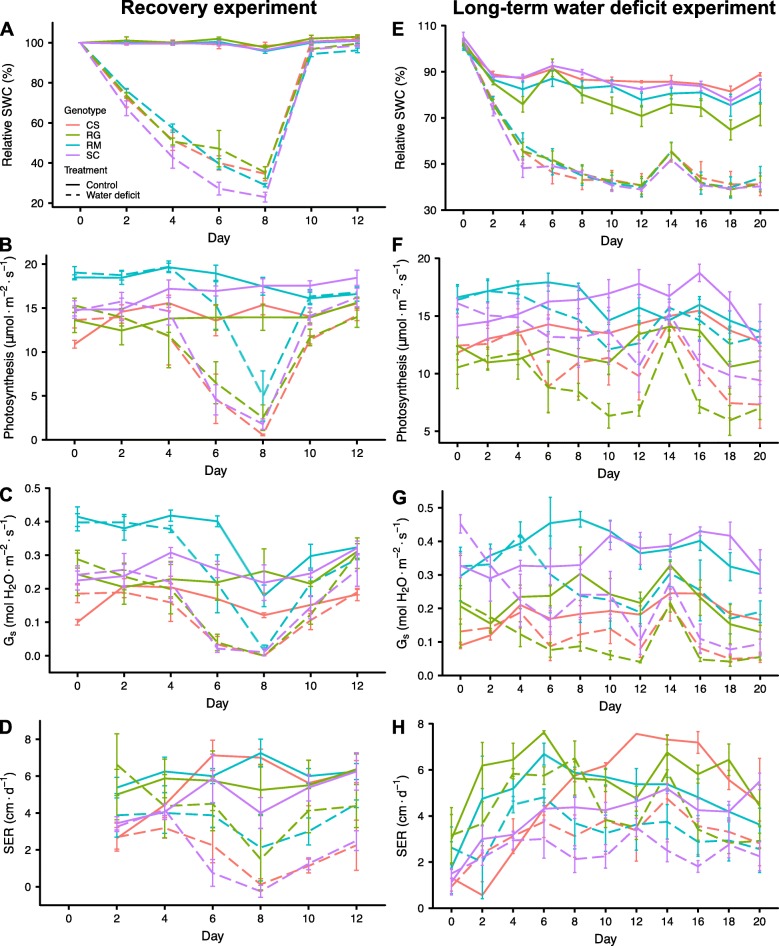
Physiological measurements of one- to three-year-old vines of the four *Vitis* genotypes during recovery and WD experiments. **a**), **e**) Relative soil water content (Relative SWC), **b**), **f**) photosynthesis, **c**), **g**) stomatal conductance (G_s_) and **d**), **h**) shoot elongation rate (SER), were measured every two days during both experiments. The first experiment consisted of a WD treatment during eight days followed by a recovery (**a**-**d**). The second experiment involved a WD treatment maintained at 50% RSWC for the stressed vines during 20 days (**e**-**h**). Red, green, blue and purple colors correspond to CS, RG, RM and SC, respectively. Data are means ± SE, n = four individual potted vines (except for RG in WD treatment with three individual potted vines for the long-term WD experiment)

**Fig. 2:**
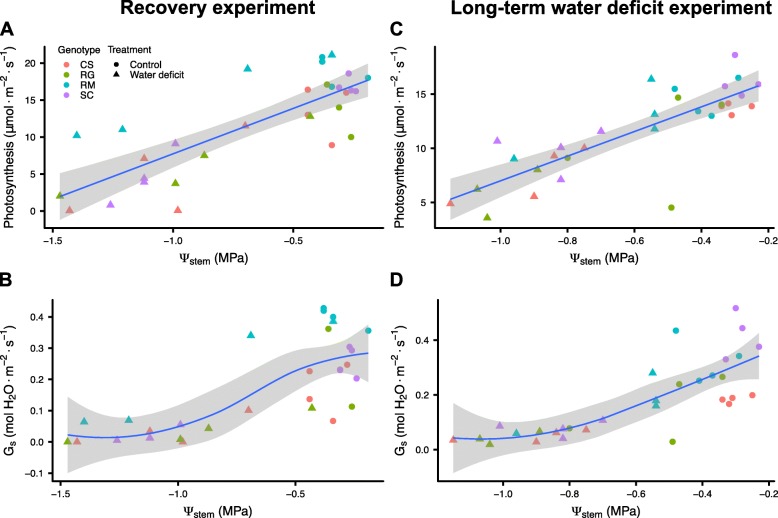
Relationship between physiological measurements of the four *Vitis* genotypes during the recovery and WD experiments. **a**), **c**) Relation between photosynthesis and stem water potential. **b**), **d**) Relation between stomatal conductance and stem water potential. The first experiment consisted of a WD treatment during eight days followed by a recovery (**a**-**b**). The second experiment involved a WD treatment maintained at 50% RSWC for the stressed vines during 20 days (**c**-**d**). Red, green, blue and purple colors correspond to CS, RG, RM and SC, respectively. Data are represented by four individual potted vines (except for RG in WD treatment with three individual potted vines)

WD experiments were performed using four different grapevine genotypes known for their contrasting drought tolerance; Cabernet Sauvignon (CS), Riparia Gloire (RG), Ramsey (RM) and SC2, a native hybrid from the Southern Nevada desert (SC). Two different WD experiments were conducted using at least one-year-old vines to elucidate the physiological responses and their relative tolerance to moderate WD for these genotypes in our greenhouse conditions. Uniform vines were grown with medium-grain sand in large tree pots. Controls were irrigated daily and WD was imposed by stopping irrigation and letting the pots dry out naturally in the greenhouse. The WD treatment was relatively equal for all genotypes; relative soil water content (RSWC) declined similarly for all genotypes with the exception of SC, which declined slightly more rapidly until day 8, when WD-treated vines were rewatered (Fig. 1a). Stem water potentials were significantly reduced by day 6 (Additional file 1) by approximately 0.6 MPa from a control value of − 0.4 MPa to a WD value of − 1.0 MPa. WD significantly reduced photosynthesis in mature leaves (Fig. 1b) by day 6, except for RM (Additional file 2), which decreased significantly two days later. The stomatal conductance of all genotypes significantly decreased by day 6 (Fig. 1c, Additional file 2). However, the stomatal conductances of CS, RG and SC were close to 0 mol H_2_O m^− 2^ s^− 1^ in WD-treated vines, whereas RM remained elevated at approximately 0.2 mol H_2_O m^− 2^ s^− 1^. Furthermore, it is interesting to note that photosynthesis was observable even when the stomatal conductance values were almost null in RM (Fig. 1b and c, day 8). The inhibition of photosynthesis was linearly related to the decline in water potential (Fig. 2a, linear model r^2^ = 0.64, p-value = 6.60e-08); the stomatal conductance response was curvilinear (Fig. 2b, cubic model, r^2^ = 0.61, p-value = 1.20e-05). It is also worth noting that the higher photosynthesis and stomatal conductance for RM corresponded to higher stem water potentials in two of the four experimental vines (Fig. 2a and b).

The shoot elongation rate (SER) was measured on a single emerging lateral shoot near the apex of a trimmed shoot/vine (see Materials and methods for more details). All other lateral shoots were removed. SER was inhibited by WD, especially that of CS and SC on day 8 (Fig. 1d, Additional file 2). Rewatering the WD-treated vines to reach 100% RSWC on day 8 allowed photosynthesis and stomatal conductance to fully recover four days later for all genotypes. However, two days after rewatering, RM had recovered more fully than SC, which had recovered more fully than CS and RG. In general, SER measurements of the one lateral shoot were much more variable than other measurements (Fig. 1d). This was in part due to our inability to control the start of the 10th lateral shoot emergence in all vines of all genotypes at exactly the same time or developmental stage. There was a lag period for growth for newly emerging shoots from the dormant bud. The more established shoots grew faster than newly emerging shoots. Eventually the younger shoots caught up and grew at similar rates. The inhibition of SER at day 8 was only slight in RM, but much greater in the other three genotypes. RG had a notable higher SER than CS or SC, whose growth had almost completely stopped. Some leaves on the lateral shoots of RG, CS, and SC were beginning to wilt on day 6, but not that of RM. The shoot apex of the lateral shoot was necrotic for three of the four WD-treated CS vines on day 8.

To investigate further the impact of moderate WD, vines were exposed to a longer stress duration at a constant RSWC in a second big pot experiment. Vines were grown for three weeks with the WD-treated vines maintained daily at 50% RSWC except at day 13, where they were watered to 60% RSWC. In accordance with the first experiment, the RSWC of WD-treated vines reached a value of 50% by the fourth day of WD (Fig. 1e). Maintenance of the vines at 50% RSWC triggered a slower decrease of photosynthesis (Fig. 1f) than in the first experiment. Stomatal conductance was significantly reduced by the 12th day of WD treatment for all genotypes (Fig. 1g and Additional file 3). At day 14, after a slight increase in water availability, all WD-treated vines were highly responsive with photosynthesis and stomatal conductance returning close to control values. Photosynthesis and stomatal conductance levels were reduced significantly on day 16 for three of the four genotypes after a return to 50% RSWC on day 15. It is noteworthy that this decrease was not observed for RM with no significant difference between control and WD-treated vines throughout the remainder of the experiment (Fig. 1f and g, Additional file 3) even though the RSWC (Fig. 1e) was reduced to the same extent as the three other *Vitis* genotypes. On the final day of WD, photosynthesis was highest for RM, which was much greater than SC, which was greater than CS or RG. This ranking is consistent with the ranking for the watering recovery responses in the first WD experiment. Stem water potentials were measured on day 18 (Additional file 4 A). Stem water potentials were higher in WD-treated RM than in the other WD treated genotypes. As in the previous experiment, photosynthesis (Fig. 2c, linear model r^2^ = 0.67, p-value = 2.22e-08) tends to be linearly related and stomatal conductance (Fig. 2d, cubic model, r^2^ = 0.63, p-value = 5.310e-06) curvilinearly-related with the stem water potential. Three of the four RM vines maintained higher stem water potentials than the other genotypes at equivalent RSWC. Root hydraulic conductivity of the vines was measured at the end of the experiment (Additional file 4 B). WD significantly reduced root hydraulic conductivity but there were no significant differences in this effect between genotypes (genotype x treatment interaction in Additional file 4 B).

**Fig. 3:**
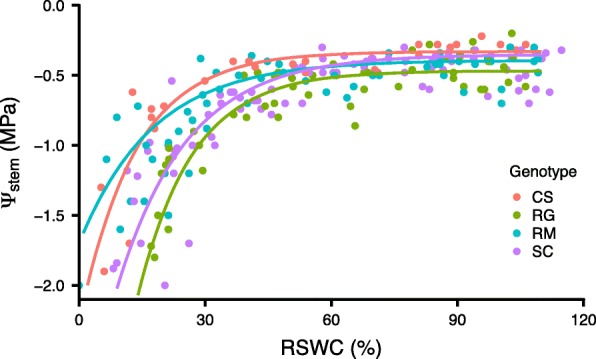
Stem water potential of the four *Vitis* species depending on the RSWC. Relationship between the stem water potential and the RSWC in the four different genotypes grown in small pots. Red, green, blue and purple colors correspond to CS, RG, RM and SC, respectively. They are represented by 27, 52, 59 and 78 individual potted vines, respectively. Non-linear regression models were predicted for each genotype using an exponential equation (One-phase association) and the corresponding regression curves were drawn

To follow up on these observations, small rooted-cuttings were grown in sand in smaller pots and allowed to naturally dry. Stem water potentials were determined at various RSWC for each genotype to determine the relationship of stem water potential to RSWC (Fig. 3). Stem water potential declined very slightly and linearly as RSWC declined between 100 and 50%. By 30% RSWC, stem water potentials declined rapidly with decreasing RSWC. Differences were small, but RM tended to decline in the rapid phase at a lower RSWC than RG, maintaining higher stem water potentials at the equivalent RSWC. Note that a similar response occurred in the big pots but at a higher RSWC (data not shown). In summary, the results from these three WD experiments indicated that RM was able to tolerate soil WD by maintaining higher photosynthesis than that of the other genotypes at the equivalent level of WD (RSWC).

### Differentially expressed genes (DEGs) were identified in moderate and severe WD in small pots

Two additional WD experiments were conducted with rooted-cuttings in the small pots. One was conducted at moderate WD (detailed below) and the other used larger plants in the same small pots to produce a more rapid and more severe WD (Additional file 5). The vines of the first small pot experiment with moderate WD were used for an extensive transcriptomic study to be discussed in more detail below. The second small pot experiment with severe stress was used to confirm specific DEGs identified in the first experiment.

### Moderate WD revealed distinct gene expression differences between genotypes

One-month-old rooted cuttings were irrigated (100% RSWC, control) or not irrigated (moderate WD) for two weeks. Rates of WD were moderate because the rooted cuttings were small and thus transpired water more slowly than larger plants. Roots and leaves from control and WD-treated vines were harvested after one and two weeks of moderate WD. Stem water potentials declined moderately in WD-treated vines (− 0.49 ± 0.04 MPa to − 0.82 ± 0.06 MPa after 1 and 2 weeks WD, respectively; mean ± SE (n = 7 individual vines)). Control vines were maintained at higher water potentials (− 0.26 ± 0.02 to − 0.32 MPa ± 0.02 after 1 and 2 weeks, respectively; mean ± SE (n = 7 individual vines)). Total RNA was extracted from the one- and two-week samples for RNA sequencing (RNA-Seq) and transcript abundance quantification (see Materials and methods for more details on data processing and quantification).

**Fig. 4:**
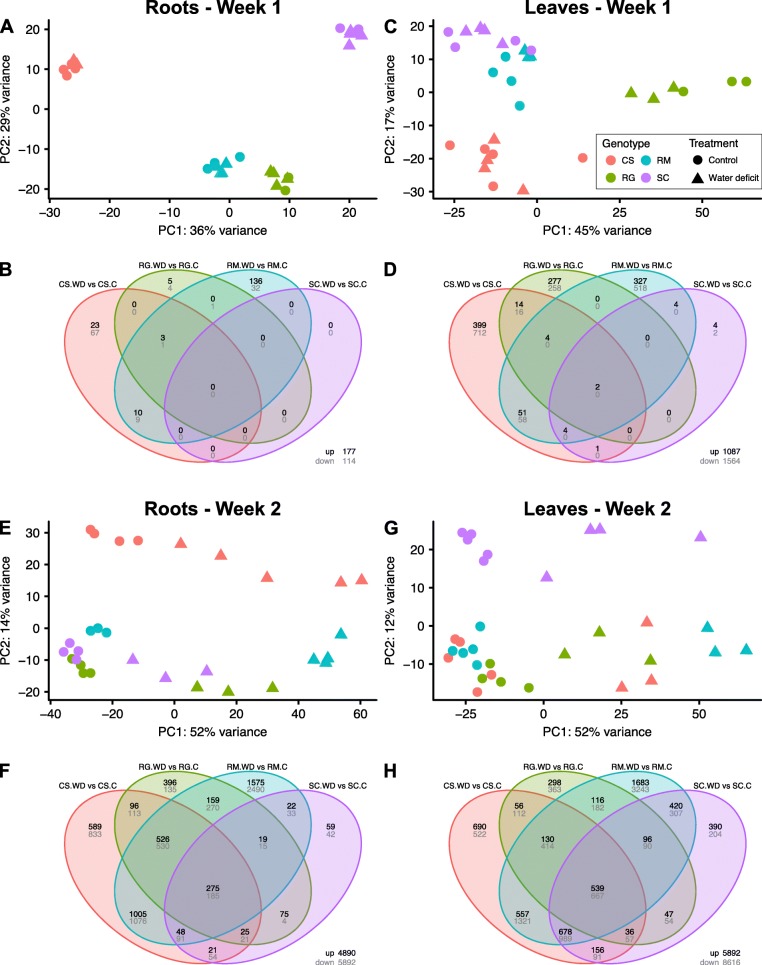
PCA and differential expression analysis results overview. **a**), **c**), **e**) and **g**) PCA representation of the samples collected from the roots or leaves after the first and the second week of treatment, respectively. Red, green, blue and purple colors correspond to CS, RG, RM and SC, respectively. Circles and triangles represent control and drought treatment, respectively. **b**), **d**), **f**) and **h**) Venn diagrams of the DEGs between drought and control vines. The color code used to differentiate the genotype is identical to that used for the PCAs

Using principal component analysis (PCA), the transcripts from root samples from week 1 were seen to be tightly separated by genotype (Fig. 4a) and this grouping pattern was similar for the leaves (Fig. 4c). The total number of genes that were differentially expressed (False discovery rate adjusted p-value < 0.05) in the roots were 291 and 10,714 and in shoots were 2604 and 14,264 in week 1 and week 2, respectively (Additional file 6). The Venn diagrams of these DEGs support the conclusion that there were different transcriptomic profiles amongst genotypes and WD treatments. Very few DEGs were shared among the four genotypes after one week of treatment (Week1; Fig. 4b and d). In addition, there were approximately nine times more DEGs in leaves as compared with roots, which is consistent with previous observations [32]. There were more DEGs in CS and RM indicating that transcriptional responses were more responsive, occurring at higher RSWC and stem water potentials compared with the other two genotypes (RG and SC). After the second week of WD, the samples showed much greater covariation than those in Week 1 (Fig. 4e and g). In the roots, the CS samples were more distinct from the three other species (Fig. 4e) while in the leaves, SC was the most distinct (Fig. 4g). Interestingly, PCA representations indicated a stronger or more distinct response to WD for RM, highlighted by pronounced sample separation. Compared with the first week, the second week triggered a significantly higher number of DEGs in response to WD (Fig. 4f, h). For all genotypes, the number of DEGs was higher for leaves than for roots. For both organs, there were more DEGs for RM, followed by CS, then RG and SC. Altogether, these results showed a well-defined separation depending on the genotype after one week of treatment, but after the second week, the WD was the leading factor for sample separation.

### Functional categories enrichment revealed an earlier transcriptomic response to moderate WD for drought tolerant species and a common core response after two weeks of treatment

**Fig. 5:**
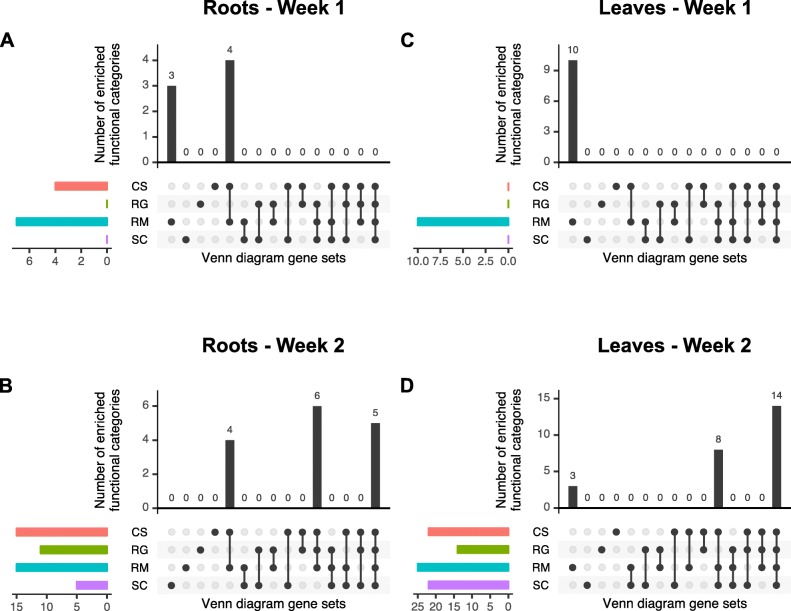
Sum of the enriched functional categories related to WD response. Enrichment analyses were performed on GO and BIN codes list extracted from the Venn diagrams of Fig. 2. The sum of the number of enriched functional categories related to ABA, response to water deprivation and galactinol synthase is represented. The vertical barplot shows the number of these enriched functional categories for the different Venn gene sets. The colored horizontal barplot shows the total number of enriched functional categories per genotype. Red, green, blue and purple colors correspond to CS, RG, RM and SC, respectively. (Details on the functional categories used can be found in the Additional file 7)

The different gene sets separated by Venn diagram representations were analyzed through gene set enrichment analyses using gene ontologies (GOs) and BIN codes (BINs). After the first week of WD, a small number of DEGs were detected in the roots, almost exclusively in CS and RM (Fig. 4b). To have a quick overview of the functional categories known to be induced by WD, the sum of the number of enriched functional categories related to ABA, response to water deprivation and galactinol synthase is illustrated in Fig. 5 (details on the functional categories used can be found in the Additional file 7). In Fig. 5a, it can be observed that three of these functional categories, representative of the WD response, were enriched for RM only, while four others were enriched in RM and CS. In total, RM showed more enriched functional categories (seven). Indeed, for this genotype both BINs and GOs related to WD response were enriched (Additional files 8 and 9; Gene list 3, Condition R1) with a significant over-representation of water deprivation and ABA related GOs in the top 5 enriched GOs and galactinol synthase for the BINs. Another BIN code was enriched corresponding to metal handling (Additional file 9, Gene list 3, Condition R1). This same functional category was enriched after one week of treatment in the leaves of RM and CS with associated GOs related to sequestering iron ion and iron ion homeostasis that were attributed to ferritin genes (Additional files 8 and 9; Gene list 11, Condition L1). As expected, after the second week of treatment, the gene sets shared by all genotypes were related to WD with ABA and galactinol synthase enriched BINs as well as response to water/water deprivation and response to ABA in the top most enriched GOs (Additional files 8 and 9; Gene list 16, Condition R2). Interestingly, these categories were the most enriched GOs in the gene set for roots shared common among all the genotypes except SC (Additional file 8, Gene list 15, Condition R2). In CS specifically, enrichment analysis showed an overrepresentation of genes involved in lipid metabolism and secondary metabolism including phenylpropanoid and flavonoid categories (Additional file 9, Gene list 9, Condition R2). Cinnamic acid related GO categories were enriched only in CS (Additional file 8, Gene list 9, Condition R2) containing cinnamyl alcohol dehydrogenase genes known to catalyze the last step of the monolignol biosynthetic pathway [33]. In RM, the most enriched GOs were related to cytoskeleton (more precisely microtubules) as well as histone H3 methylation (Additional file 8, Gene list 3, Condition R2). They were also enriched in the gene set in common with RG, with cytokinetic process in the most enriched GO category (Additional file 8, Gene list 7, Condition R2).

In the leaves, WD-related functional categories were specifically enriched in RM after one week of treatment (Additional files 8 and 9; Gene list 3, Condition L1). After the second week, galactinol synthase, ABA and lipid metabolism categories were enriched in all genotypes (Additional files 8 and 9; Gene list 16, Condition L2). In CS, flavonoid-, ABA- and fatty acid-related genes were enriched (Additional file 9, Gene list 9, Condition L2). Flavonoid and pigment biosynthetic process, ABA and response to UV-related genes were enriched in the gene set shared between CS and SC (Additional files 8 and 9; Gene list 10, Condition L2). Altogether, the gene set enrichment analyses revealed that the four genotypes shared a common core WD response. This core WD response included ABA biosynthesis/signaling and MYB transcription factors (TFs) in the roots, lipid metabolism and abiotic stress (heat) in the leaves and galactinol synthases related functional categories for both organs for all genotypes. Moreover, WD-related functional categories were specifically enriched after one week of WD in the species known for its higher drought tolerance, RM.

### ABA-related DEGs were induced earlier and stronger in RM

**Fig. 6:**
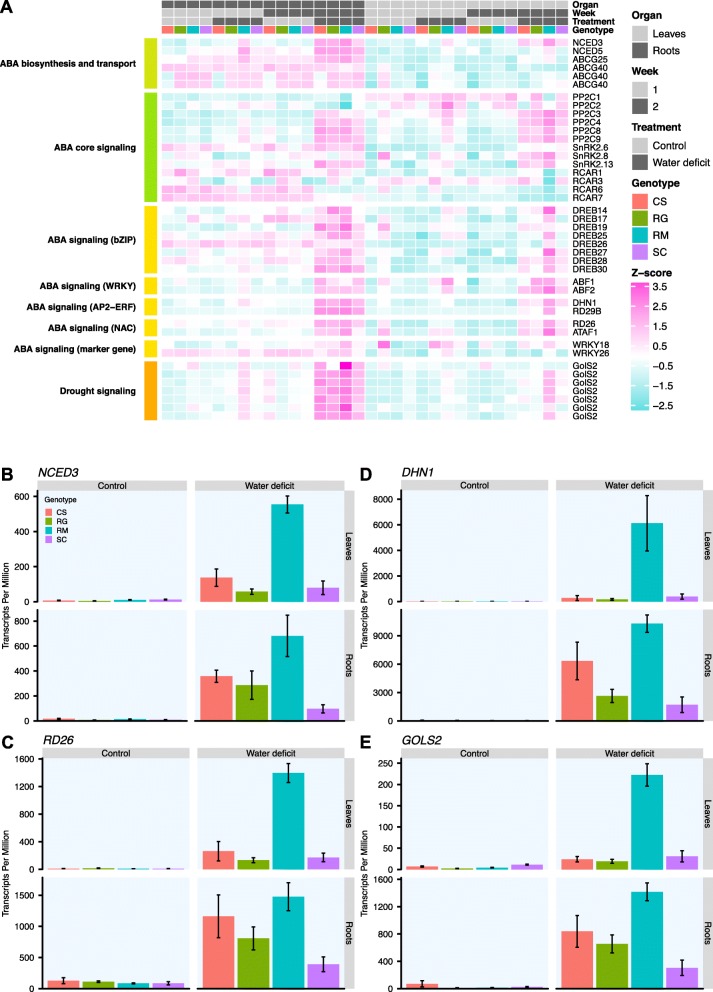
ABA-related genes expression in the four genotypes in response to water deficit. **a**) Heatmap representation of the gene expression of ABA-related genes across the different conditions. For each condition (Organ x Week x Treatment x Genotype), an average TPM value was calculated and log2 transformed. These expression values are represented as Z-scores (calculated per gene) on the heatmap and are colored from turquoise (low value) to pink (high value). Genes were clustered by process or protein family labeled on the left. At the top of the heatmap, a chart identifies the different conditions with leaves and roots in light grey and dark grey, respectively; week 1 and week 2 in light grey and dark grey, respectively; control and drought treatment in light grey and dark grey, respectively; and the genotypes CS, RG, RM and SC in red, green, blue and purple, respectively. Expression profiles of *NCED3* (**b**), *RD26* (**c**), *DHN1* (**d**) and *GOLS2* (**e**) after two weeks of treatment. Expression in control (left column) and WD treated vines (right column) for the leaves (top row) and the roots (bottom row) is represented in transcripts per million, mean ± SE, n = three-five individual vines. Genotypes are color coded as for the heatmap (**a**)

ABA-related genes represent good drought stress markers. DEGs identified in WD vs control vines at day 7 (week 1) (Fig. 6a and Additional file 10) were only significant for RM. Among them, genes involved in ABA biosynthesis, namely *NCED3* and *NCED5,* were induced by WD in the roots; *NCED3* was also a DEG in the leaves. This regulation of ABA biosynthesis genes was accompanied by a higher expression of *PP2C9* (a protein phosphatase 2C involved in ABA signaling) in both RM WD-treated organs. In the WD-treated leaves, the transcript abundance of the ABA receptor, *RCAR7,* was decreased, while that of the ABA-induced protein kinase gene, *SnRK2.13* was increased. After an additional week of WD (week 2), the transcript abundance of *NCED3* was increased in both organs in all genotypes and it was significantly higher in RM than the other genotypes (Fig. 6b). With a lower expression level, the transcript abundance of *NCED5* was higher in the WD-treated roots of all genotypes, but only in the WD-leaves of RM. The transcript abundance of ABA transporter genes for *ATP BINDING TRANSPORTER CASSETTE G 25* (*ABCG25*) was higher in the WD-treated roots (except for SC) and in the WD-treated leaves (Fig. 6a, Additional file 10). Gene expression of REGULATORY COMPONENTS OF ABA RECEPTORS (RCARs) was mostly lowered by WD (Additional file 10). In the leaves, the transcript abundance of *RCAR1*, *RCAR3*, *RCAR6* was specifically decreased for RM (Additional file 10). For all of these genes, a higher average expression was found in the WD-treated root samples compared to the WD-treated leaves, except for *RCAR6,* presenting a similar expression pattern (Fig. 6a). The gene expression of *PP2C1* and *PP2C2* was decreased in WD-treated roots and leaves, respectively, only in RM (Additional file 10). The transcript abundance of *PP2C3*, *PP2C4*, *PP2C8* and *PP2C9* was higher in the four WD-treated genotypes in both organs, except for *PP2C4* in SC WD-treated leaves. In both organs, *PP2C8* and *PP2C9* were the two PP2Cs with the highest average expression in the four genotypes with a notable higher level in the RM leaves (Additional file 10). The expression of *SNF1-RELATED PROTEIN KINASE 2* (*SnRK2*) genes was also significantly modified with a transcript accumulation of *SnRK2*.*6* in RM roots and leaves as for SC. *SnRK2.8* was differentially expressed in roots except for SC and in leaves except for RG. The transcript abundance of *SnRK2.13* was increased in both organs by WD for all genotypes. Altogether, these results showed that the transcriptional regulation of genes related to the ABA pathway was more sensitive for RM occurring at the same RSWC as the other genotypes. Moreover, even if several genes of this metabolic pathway were significantly differentially expressed after the second week of WD in the other genotypes, the expression differential remained particularly more pronounced for RM at equivalent RSWC.

### Transcriptional response of ABA-signaling transcription factor genes

The transcript abundance of ABA-signaling TFs such as ABRE-BINDING FACTORS (ABFs) was modified by WD (Fig. 6a and Additional file 10). At day 7, the transcript abundance of *ABF1* and *ABF2* was higher in the RM leaves only. Some WRKY TFs, *WRKY18* and *WRKY26,* were modified with the same expression pattern. The second week of treatment triggered the transcriptional regulation of many more TFs. While *ABF2* transcript abundance was higher in both organs of all genotypes, this was only true for *ABF1* in CS and SC roots and for all genotypes except SC in the leaves (Additional file 10). The transcript abundance of *WRKY18* was increased in RM roots only. In addition, the expression of many APETALA2-ETHYLENE RESPONSIVE FACTOR (AP2-ERF) TFs was affected. Most of the DEGs found among DEHYDRATION RESPONSE ELEMENT-BINDING (DREB) TFs in roots were specific for RM, except *DREB19* and *DREB30,* which were also DEGs for CS and RG (Additional file 10); similar trends were observed in leaves. Gene expression was consistently higher in RM particularly for *DREB14*, *DREB17*, *DREB25*, *DREB26*, *DREB27* and *DREB28*. NAC DOMAIN CONTAINING (NAC) TFs showed similar responses, with an induced expression of *RESPONSIVE TO DESICCATION 26* (*RD26*) and *ARABIDOSIS THALIANA ACTIVATING FACTOR 1* (*ATAF1*)*,* particularly in RM (Fig. 6c). Other ABA-marker genes were highly-induced DEGs, notably *RD29B* and *DEHYDRIN 1* (*DHN1*). Interestingly, the expression of the *DHN1* was strongly induced in RM organs, with a Transcripts Per Million (TPM) value above 10,000 in roots as well as an average expression at least 16-times higher than the other genotypes in leaves (Fig. 6d). As expected, WD treatment also affected several genes involved in drought responses. Most of them were DEGs during the second week. However, *GALACTINOL SYNTHASE 2* (*GOLS2*) gene expression was higher specifically in RM at day 7. The second week of treatment triggered the transcript accumulation of *GOLS2* orthologs in both organs of most of the genotypes with a higher increase in RM (Fig. 6e and Additional file 10).

These results confirmed that the higher expression of ABA biosynthesis genes in RM was accompanied by a higher transcript accumulation of ABA-signaling genes. These results observed on one-month-old cuttings were also observed in the five-week-old vines which experienced a stronger stress (stem water potentials were approximately − 1.5 MPa) after one week of treatment (Additional file 5). Gene expression profiling using RT-qPCR was performed on *NCED3* and *DHN1* (Additional file 11). These two genes showed a strong upregulation in response to WD in RM in the RNA-Seq results after two weeks of treatment. The closest Arabidopsis orthologs of *DHN1* are known ABA markers, *RAB GTPASE 18* (*RAB18*) and *DEHYDRIN XERO 1* (*XERO1*). Interestingly, the expression of *NCED3* was significantly increased in RM roots in response to WD and in RM and CS leaves (Additional file 11A). *DHN1* expression was also induced specifically in RM roots and showed a significantly stronger expression in the leaves in this genotype in response to WD (Additional file 11B) while these plants were at the same RSWC (Additional file 5A). Again, these results confirm that the ABA-related gene network seems more dynamically regulated in RM in response to WD.

### Major intrinsic proteins (MIPs) were differentially expressed in WD vines

**Fig. 7:**
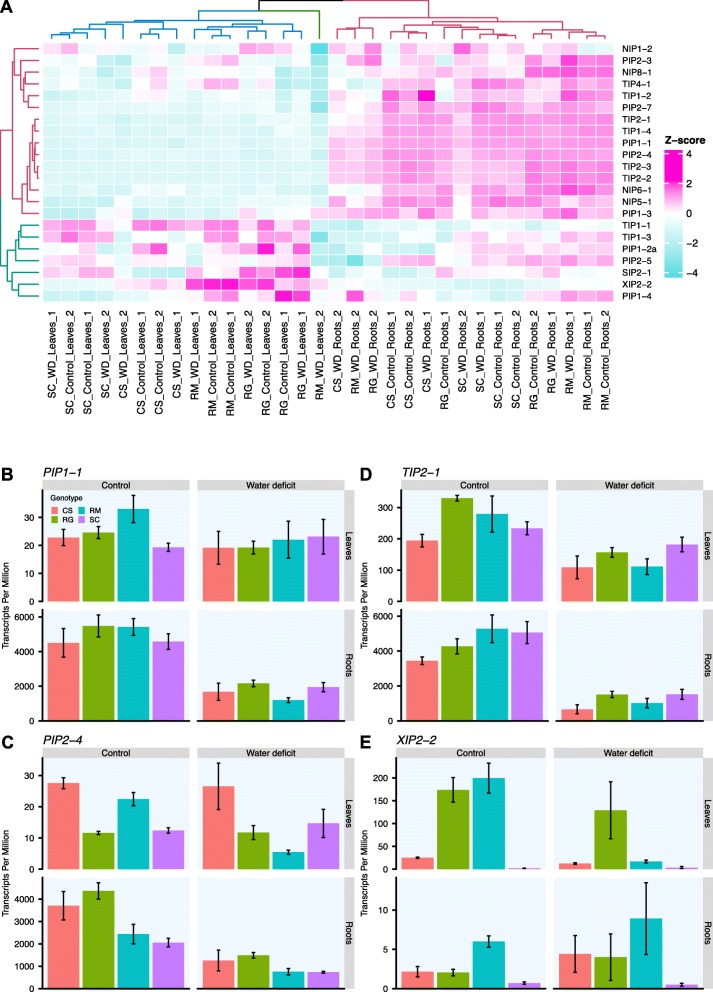
MIP-related genes expression in the four genotypes in response to water deficit. **a**) Clustering of the MIP family members. The heatmap represents transcript abundance of the MIP family genes, log2 transformed TPM values are represented as Z-scores calculated per gene ranging from turquoise to pink color for low to high values. Dendrograms were colored to distinguish the main clusters. Expressed genes were selected by removing genes with log2TPM < 1 in more than 75% of the samples. Expression profiles of *PIP1–1* (**b**), *PIP2–4* (**c**), *TIP2–4* (**d**) and *XIP2–2* (**e**) after two weeks of treatment. Expression in control (left column) and WD treated vines (right column) for the leaves (top row) and the roots (bottom row) is represented in transcripts per million, mean ± SE, n = three-five individual vines. The genotypes CS, RG, RM and SC are represented in red, green, blue and purple respectively

The MIP family proteins that facilitate water and small molecule transport across membranes are represented by 29 members in grapevine [34]. Cluster analysis of the expressed MIP genes revealed a clear separation by organs with two main groups presenting opposite expression patterns; some genes were highly expressed in roots and lowly expressed in leaves or vice versa (Fig. 7a). A third cluster represented WD-treated leaf samples for RM at week 2 for which most MIP genes had low transcript abundance. Amongst all *Vitis* MIPs, only *PLASMA MEMBRANE INTRINSIC PROTEIN 1–3* (*PIP1–3*) was induced by WD relative to control plants (Additional file 12); it was significantly increased in WD leaves of SC and RM at week 2. Only two DEGs, *PIP1-2a* and *TONOPLAST INTRINSIC PROTEIN 1–3* (*TIP1–3*), were decreased by WD specifically in RM leaves at day 7. Amongst the PIPs, it should be noted that *PIP1–1* had the highest expression and appeared to be root specific with an average TPM expression at least 100 times higher than in the leaves (Fig. 7b). *PIP2–4* gene expression was also high in the roots and was decreased by the WD treatment in all genotypes (Fig. 7c and Additional file 12). *PIP2–5* had similar expression pattern except that the decrease was not significant for SC. Interestingly, these two genes, as well as *PIP2–7* showed an expression decrease by WD only in RM leaves. Amongst the TIPs, *TIP2–1* (Fig. 7d) and *TIP1–4* had the highest expression, particularly in roots with a significant decrease in all genotypes by WD, except for SC for the latter gene (Additional file 12). Lastly, *X INTRINSIC PROTEIN 2–2* (*XIP2–2*) expression was detected mainly in RM organs and mostly in leaves (Fig. 7e). Its expression, not detectable for CS and SC, was induced in both control and WD treatment in RG, while it was significantly repressed in RM leaves by the WD.

### ABA biosynthesis and lipid metabolism were common pathways responding to WD

**Fig. 8:**
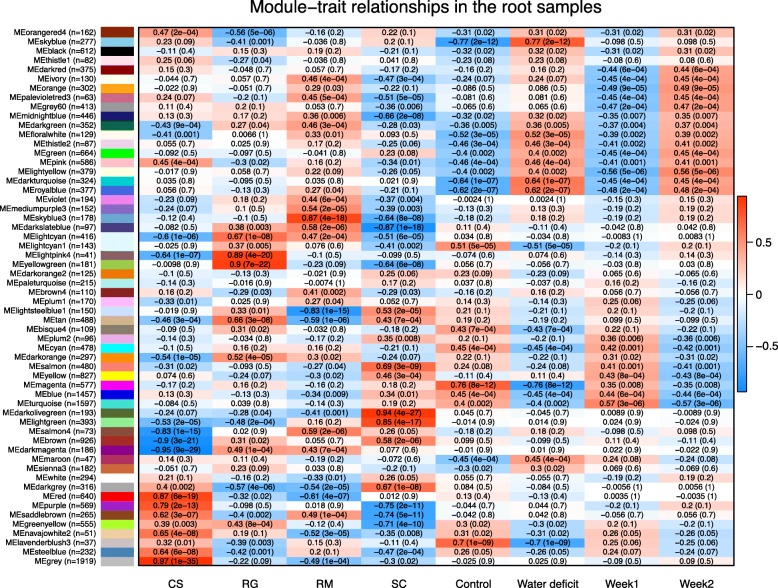
WGCNA on the root transcriptome. Correlation heatmap among the identified modules and the different experimental conditions for the root samples. Experimental traits are presented in columns and their association with module eigengene (rows) is represented by a Pearson’s correlation coefficient and a p-value within parentheses. The color of each cell ranges from blue indicating a high negative correlation to red for a high positive correlation. The number of genes included in each module is presented within parentheses

**Fig. 9:**
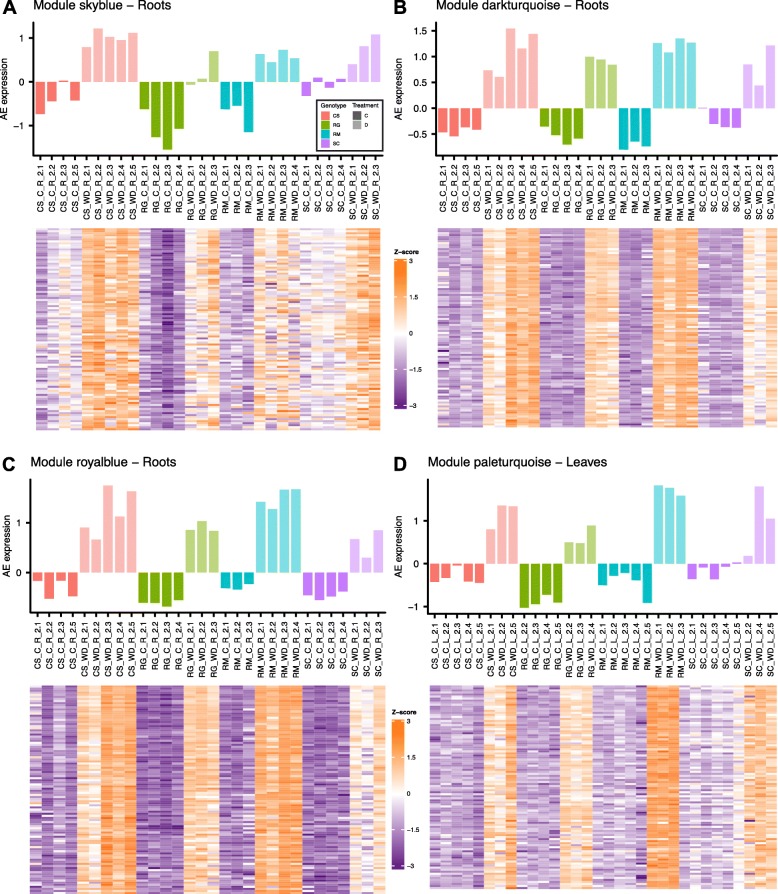
Gene expression in WGCNA modules. Eigengene average expression for the root modules “skyblue” (**a**), “darkturquoise” (**b**), “royalblue” (**c**) and for the leaf module “paleturquoise” (**d**) after two weeks of treatment. Samples are represented in columns. Red, green, blue and purple colors correspond to CS, RG, RM and SC, respectively. Solid and semi-transparent colors correspond to control (**c**) and water deficit (WD) treatments, respectively. Lower heatmaps represent Z-scores calculated per gene using log2 transformed TPM values of the top 100 most connected genes in the module (orange indicates high expression and purple low expression)

In response to WD, a plethora of genes was transcriptionally regulated in the four grapevine genotypes. In order to identify genes associated with drought responses in all four *Vitis* species and to identify potential species-specific gene subnetworks, a weighted gene co-expression network analysis (WGCNA) was performed separately for both organs to better define modules (e.g. gene clusters). In roots, 58 distinct modules were identified (Fig. 8). To determine how a given gene relates to a specific module, a correlation was calculated between its expression profile and the module eigengene (Additional file 13). A module eigengene is an artificial gene considered as a representative of the gene expression patterns in a module [35]. Based on the correlation to the module eigengene, the top 100 most highly correlated genes from each module were extracted and an enrichment analysis of the GO functional categories was performed (Additional file 14). Fourteen of these modules were positively correlated and ten were negatively correlated with WD at p-value < 0.05. Most of the positively correlated modules were enriched in the response to water deprivation GO category (Additional file 14). Gene set enrichment analyses of the negatively correlated modules showed enrichment for numerous processes related to the regulation of growth. Three major modules were the most positively correlated with WD (Pearson’s correlation coefficient > 0.6) (Fig. 8): skyblue (Fig. 9a), darkturquoise (Fig. 9b) and royalblue (Fig. 9c). The top 100 most connected genes for each of these modules presented a tightly conserved expression pattern across the samples (heatmaps in Fig. 9). The skyblue module was enriched in response to water deprivation, response to acid chemical, and lipid metabolism-related genes; it was negatively correlated with drought-sensitive RG with a low average eigengene expression after WD treatment (Fig. 9a). Amongst the top 150 genes that were most correlated in this module, gene orthologs encoding GLYCEROL-3-PHOSPHATE ACYLTRANSFERASE (GPAT7), 3-KETOACYL-COA-SYNTHASE 1 (KCS1), GDSL-MOTIF ESTERASE/ACYLTRANSFERASE/LIPASE LIKE (GDSL-like), LIPID TRANSFER-LIKE PROTEIN VAS (VAS), BETA-KETOACYL REDUCTASE 1 (KCR1), FATTY ACID DESATURASE 5 (ADS3), CYTOCHROME P450 FAMILY 86 SUBFAMILY 86 POLYPEPTIDE 1 (CYP86A1), CASP-LIKE PROTEIN 1D1 (CASPL 1D1), GPAT4, ACYL-ACTIVATING ENZYME 3 (AAE3), SULFOQUINOVOSYLDIACYLGLYCEROL 1 (SQD1), SQD2 were identified. Gene set enrichment analysis for the darkturquoise module indicated that this module was related to water deprivation, ABA and galactinol synthase categories. *RD29B* (LTI65) was most connecteded gene within this module and is an ABA-induced marker gene. Among the other highly connected genes (top 100) in this module, were several other ABA-related genes such as *DHN1*, *HIGHLY ABA-INDUCED PP2C GENE 2* (*HAI2*), *ABA INSENSISTIVE 1* (*ABI1*, also known as *PP2C4*), *ABF2*, *DREB2A*, *NCED3* and *CYP707A2*. It should be noted that the lightyellow module, positively correlated with WD, was also a gene cluster for which the hub gene (most connected gene to this module) was a NAC domain containing protein, *RD26/NAC072*. Other ABA-related genes were also found in this module. The enriched functional categories for the darkturquoise module were representative of a WD response (Fig. 9b). The genes highly connected to the royalblue module were also closely related to ABA (Fig. 9c and Additional file 14). *ABI1* is the gene that was most connected in the royalblue module, closely followed by *LATE EMBRYOGENESIS ABUNDANT 7* (*LEA7*), *NCED3* and *GOLS2* (within the top 5 most-connected genes). The thistle2 module, significantly correlated with WD, had heat responsive genes over-represented. Indeed, the top 25 genes connected in this module involve 21 genes corresponding to heat shock proteins (HSPs) along with two putative orthologues of *AtGOLS1*, a UBX domain-containing protein and an unknown protein. The GO categories enriched in this module included response to ABA, response to alcohol and response to lipid. The skyblue3 module was most positively correlated with RM and negatively correlated with SC. This module was enriched in cysteine biosynthesis, serine and glutathione metabolism. Regarding the recent findings about cysteine and ABA biosynthesis [28], it is noteworthy that in the most connected genes in this module (top 30), there was, for example, a gene putatively encoding a *SERINE ACETYLTRANSFERASE 3* (*SAT3*) known to catalyze the first step of cysteine biosynthesis from serine (Additional file 15). It was more expressed in RM roots than the other genotypes and the expression differential was higher for RM in response to WD.

**Fig. 10:**
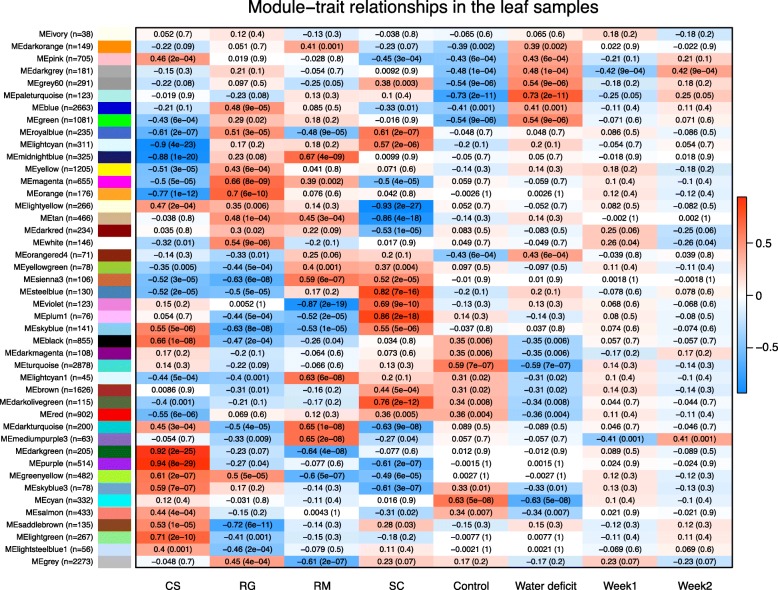
WGCNA on the leaf transcriptome. Correlation heatmap among the identified modules and the different experimental conditions for the leaf samples. Experimental traits are presented in columns and their association with module eigengene (rows) is represented by a Pearson’s correlation coefficient and a p-value within parentheses. The color of each cell ranges from blue indicating a high negative correlation to red for a high positive correlation. The number of genes included in each module is presented within parentheses

WGCNA of leaf samples provided additional insights into the transcriptomic responses to WD (Fig. 10 and Additional files 16 and 17). There were eight positively correlated and ten negatively correlated modules with WD. In general, positively correlated modules were enriched in response to water deprivation, response to ABA and response to lipid genes and negatively correlated modules were enriched in photosynthesis, growth, plastid and cell wall organization genes. The paleturquoise module in leaves (Fig. 9d) was most positively correlated with WD. Like the roots, several ABA-related genes such as *NCED3*, *PP2C*, *ABI2*, *ABF2*, *RD22* and *GOLS2* were in the top 100 genes most correlated within this gene cluster and several HSPs clustered together in the leaves in the darkgrey module.

### Strong DEGs involved in the common core response to WD in the four genotypes are more responsive in RM

**Fig. 11:**
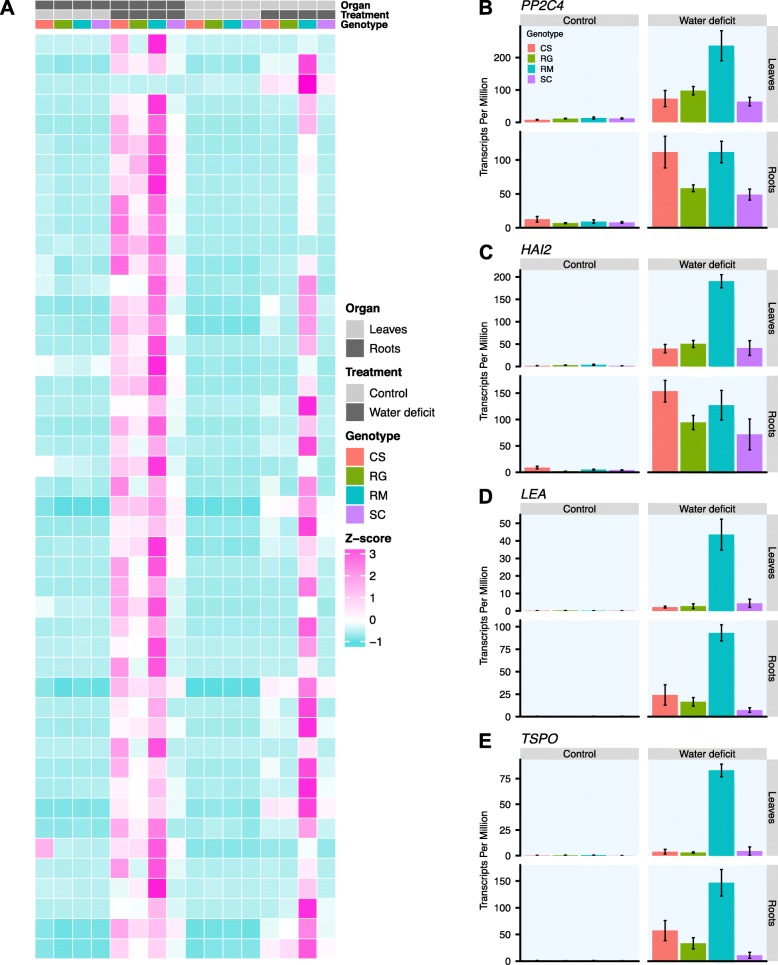
Expression profiles of DEGs induced by the WD in roots and leaves after two weeks of treatment in RM. **a**) Heatmap representation of the gene expression across the different conditions after two weeks of treatment. For each condition (Organ x Treatment x Genotype), an average TPM value was calculated and log2 transformed. These expression values are represented as Z-scores (calculated per gene) on the heatmap and are colored from turquoise (low value) to pink (high value). At the top of the heatmap, a chart identifies the different conditions with leaves and roots in light grey and dark grey respectively, control and water deficit treatment in light grey and dark grey respectively and the genotypes CS, RG, RM and SC in red, green, blue and purple respectively. Expression profiles of *PP2C4* (**b**), *HAI2* (**c**), *LEA* (**d**) and *TSPO* (**e**) after two weeks of treatment. Expression in the control (left column) and WD treated vines (right column) for the leaves (first row) and the roots (second row) is represented in transcripts per million (TPM), mean ± SE, n = three-five individual vines. The different genotypes are represented using the same color code used for the heatmap (**a**)

For each genotype, sets of DEGs were selected for being strongly upregulated in both roots and leaves by the WD (showing a LFC > 4 in both organs in response to WD). Such DEGs were detected only for the second week of treatment with 19, 20, 36, 143 DEGs for RG, SC, CS, and RM, respectively. These gene lists were then filtered to detect expressed genes which were significantly more induced in a given genotype compared with the three others. After this step, only one gene was detected in CS, but it was hard to draw any conclusion due to the biological variability observed for this gene; after filtering, no gene remained for RG and SC. The only strikingly significant gene list identified corresponded to the RM genotype containing 46 DEGs. These genes were strongly induced in both roots and leaves (LFC > 4) after two weeks of WD treatment and were significantly more expressed than in the three other genotypes (Fig. 11a). Some of these genes were included in the common core gene set in response to WD shared by the four genotypes, but they were induced to a much lower extent in the shoots or not expressed at all in the three other genotypes relative to RM. Gene set enrichment analysis identified response to water deficit and ABA as the top enriched functional categories for this gene set (data not shown) including *PP2C4* (*ABI1*) (Fig. 11b) and *HAI2* involved in ABA signaling (Fig. 11c). In addition, a *LEA* and a *TSPO RELATED PROTEIN* (*TSPO*) gene known to be involved in the response to ABA (Fig. 11d and e) were in this gene set. Several other genes known to be involved in WD response were also included in this list (Additional file 18) as well as a great number of genes of unknown functions. Also, it has been previously shown that *ABI5* expression, another ABA and dehydration responsive transcription factor, is induced by a rapid and severe dehydration, particularly in RM leaves as compared to RG [12]. Interestingly, one gene encoding an EARLY METHIONINE-LABELLED 6 PROTEIN (GEA6) (*VIT_13s0067g01240*), known to be activated by the direct binding of ABI5 to its promoter, was also found in this gene set. Its expression was strongly induced in RM leaves exclusively in response to WD. This further confirms the potential importance of *ABI5* in drought tolerance. Together, these results indicated that WD induced a stronger modification of the transcriptome in RM leaves. Even at the same expression level in the roots of the four genotypes, gene expression in leaves for several drought related genes was significantly higher for RM in response to WD. As RM was the most drought tolerant among the four *Vitis* genotypes studied, these genes responding to potential root-to-shoot signals from WD may significantly participate in its drought tolerance.

## Discussion

### RM had distinct physiological and molecular strategies to cope with WD

Under the greenhouse conditions of this study, we determined that RM was the most tolerant to WD compared to the three other grapevine genotypes. In old plants grown in large pots, major factors associated with WD tolerance were identified to be maintenance of higher stomatal conductance and photosynthesis during WD. RM kept its stomates open for a longer period and maintained higher rates of photosynthesis. Higher stomatal conductance was associated with higher drought tolerance for RM in another study as well [30]. RM recovered more quickly after WD was removed and became more insensitive to subsequent dehydration. These physiological responses presumably improved its energy status relative to RG, the most drought sensitive genotype of this experiment. Concerning the stem water potential, the only experiment designed to measure this property in relation to the pot RSWC was illustrated in Fig. 3. More replications of this experiment need to be performed to better capture potential genotypic differences during the decrease of stem water potential at low RSWC (from 50 to 0%); the (an) isohydric nature of the plant known to be directly associated to its drought tolerance. The other experiments included in this paper do not allow any further conclusions regarding this parameter since the plants were either harvested before or after the stem water potential drop. RM is known to develop a deeper rooting depth [30] and smaller leaves [36] compared with RG, which may contribute to a higher water status in the vine, allowing this photosynthetic strategy to succeed under these conditions. The higher energy status may also contribute to enhanced root growth. In younger plants grown in smaller pots, RM had a larger transcriptomic response including ABA- and MIP-related genes known to influence water relations. RM had higher *NCED3* transcript abundance in response to WD and presumably higher ABA concentrations in the leaves in the young vines. *NCED3* expression is sensitive to the cysteine concentration [28] and this too may have been enhanced in RM. This study [28] shows that there are several factors (sulfate, cysteine biosynthesis, etc.) that can influence stomatal conductance by way of ABA biosynthesis; however, ABA is still considered a major regulator during WD. In a previous study [36], the stomatal conductance of RG was much more sensitive to rapid dehydration than that of RM, yet both species showed similar sensitivity to applied ABA. It remains to be determined if stomatal sensitivity to ABA changes with the duration of WD. RNA-Seq and RT-qPCR performed on these vines both showed the same strong upregulation of genes involved in response to WD that were more enhanced in RM. This suggests a more dynamic transcriptome regulation when the soil water content is limiting for this genotype. RM may be better adapted to WD because it evolved in the hot, dry climate of Texas in the southern United States, whereas RG evolved in the cool, wet climate of the North Eastern United States. Altogether, it is interesting to note that the stronger drought tolerance of RM is associated to multiple mechanisms across developmental stages. Indeed, in early stages of growth, RM is the most responsive genotype to the WD presenting a stronger transcriptomic regulation compared with the three other genotypes. Moreover, in older vines, this genotype evolved unique adaptive strategies allowing a higher drought tolerance.

### ABA biosynthesis and signaling was a core response to WD in grapevine

The transcriptomic analysis revealed many genes that increase and decrease in transcript abundance in response to WD in the four grapevine genotypes by WD (Fig. 4). However, it should be noted that reads from all species were aligned against the *Vitis vinifera* reference genome [37]. A stronger distinction between genotypes would likely have been observed if reads were aligned against their own genomes or de novo transcriptome assemblies specific for each species. Such a strategy may require a deeper sequencing depth than the one used in the present study. Another note of caution concerns gene duplication. Indeed, a high transcript abundance for a gene could be explained by a strong gene duplication in a specific genome. As expected, DEGs were mostly related to water deprivation response and response to ABA. In addition to the *NCED* (ABA biosynthesis) genes, the expression of key players of the ABA core signaling pathway such as *PP2Cs*, *SnRK2s* and *ABFs* was significantly modulated by the WD treatment in all genotypes (Fig. 6a and Additional file 10). An extensive analysis of the transcriptomes of the four *Vitis* species using WGCNA confirmed the differential expression analysis results identifying conserved gene modules amongst genotypes that seem to play a crucial role in response to water deprivation (Fig. 8). In the roots, the ABA-related modules darkturquoise and royalblue identified two hub genes, *RD29B* and *ABI1*, the most connected genes in these modules, respectively. *RD29B* is described as an important actor of the drought transcriptional memory response [37﻿] with a gene expression induction strongly promoted by recurrent drought stresses. In perennials, memory stress actors seem to be even more indispensable due to the acclimation requirement to cope with repetitive stresses across years [38﻿]. Moreover, the epigenetic nature of this mechanism reinforces its relevance in understanding *Vitis* genotypic variation for drought tolerance [39, 40]. Furthermore, it has been shown that previous exposure to WD in a grapevine lifecycle influences the regulation of its stomatal aperture [41]. *ABI1* encodes a PP2C phosphatase that negatively regulates ABA signaling [42]. It is the most highly connected gene in the royalblue module (Pearson’s correlation coefficient 0.984) and is closely followed by other important ABA- and drought-related genes including *NCED3* (0.974). It is interesting to find both positive and negative actors involved in the ABA pathway that were closely connected. This suggests a very tight regulation of this pathway. This module seems to represent the regulatory core cluster of the ABA-dependent drought response in *Vitis*. The strong correlation of *NITRATE TRANSPORTER 1/PEPTIDE TRANSPORTER FAMILY 6.4* (*NPF6.4*) in this module and its strong gene expression induction in response to drought indicates its potential involvement in ABA transport like the previously described NPF4.6 [43]. The closest orthologs of *AtNPF4.6* in the Pinot Noir genome are *VIT_01s0026g01570* and *VIT_17s0000g05640*; their expression profiles, however, were not highly correlated to *NCED3* contrary to the two orthologs of *NPF6.4* (*VIT_02s0087g00580* and *VIT_12s0059g01240*). The module skyblue was also significantly correlated with WD and the analysis of the genes highly connected to this module indicated that it was related to lipid metabolism. It represents a promising cluster to explore genes involved in lipidic barrier formation such as the suberin deposition process, an important feature for drought tolerance [44].

### The antioxidant role of ABA in drought tolerance

In addition to the regulation of stomatal conductance, ABA has multiple roles related with dehydration responses. ABA regulates seed germination, cell and organ growth, energy metabolism and the production of antioxidants and osmoprotectants [19, 45, 46]. For example, the transcript abundance of galactinol synthases is highly responsive to WD [47] and ABA [47, 48]. Over-expression of galactinol synthases in plants grown in the lab or in the field leads to significant increases in drought tolerance resulting in increased yields [47, 49]. It is thought that the downstream products (raffinose family oligosaccharides) of these enzymes act as antioxidants and osmoprotectants resulting in better photochemical efficiency in the leaves [47]. Both the expression of *NCEDs* and galactinol synthases were highly responsive to WD in RM and expressed at higher levels. In addition, the constitutive expression of genes involved in glutathione metabolism was higher in RM. The ascorbate-glutathione cycle is very important as an antioxidant defense [50].

### Responses of MIPs to WD

With an important role in water transport, aquaporins (AQPs), belonging to the MIP family, represent good candidates to investigate genotype variation in drought tolerance. Furthermore, ABA appears to affect the transcript abundance and activities of some AQPs [51]. AQPs were suggested to play a key role in maintaining water homeostasis in response to environmental stress conditions [52, 53]. The pattern of expression of genes encoding AQPs in response to drought stress remains equivocal. While some studies indicate upregulation of AQPs to facilitate water transport during WD, other researchers highlight their downregulation to hinder excessive loss of water [54]. This highlights the variability of AQP’s gene expression and WD responses depending on the cell tissue, organ or genotype and the severity and duration of the WD. In response to 12 days of WD, the expression of most of the AQP genes is reduced in Arabidopsis, supposedly to reduce the water flow through cell membranes and avoid further loss of leaf turgor [55]. The analysis of the gene expression of the MIP encoding genes in the four *Vitis* species in response to WD revealed a similar pattern with a strong decrease in RM leaves (Fig. 7). In the roots, after two weeks of WD, the response tended to be similar amongst genotypes with a reduced expression compared with well-watered vines. This indicates that RM is more efficient at regulating actively AQPs to maintain water homeostasis and this may have contributed to its higher stem water potentials and drought tolerance. While these genes were expressed in the roots of the four genotypes, the shoot response seemed to be stronger for RM indicating a more efficient and sensitive communication from the roots, where the drought perception occurs, to the shoot, where the regulation of the transpiration and photosynthesis takes place. This presumes that surface roots begin to dehydrate and send signals to the shoot before any noticeable change in water status of the shoot.

### Root to shoot long-distance signaling is a key mechanism to tolerate drought stress

Rootstocks play a critical role in the adaptation of the scion in response to water shortage [56]. However, mechanisms allowing rootstocks to cope with water stress to support the harmonious growth of the scion-producing berries are still largely unknown. Takahashi et al., [57] recently described a long-distance mechanism to control stomatal aperture in response to WD. Drought stress perception in the roots induces the expression of *CLAVATA3/EMBRYO-SURROUNDING REGION-RELATED 25* (*CLE25*) in the roots [57]. CLE25 peptides are transported to the leaves where they are perceived by BARELY ANY MERISTEM 1 and 3 (BAM1 and 3) receptor-like kinases. This long-distance peptide transport induces an increase of ABA levels via the induction of NCED3 and triggers stomatal closure. The closest orthologous genes of *CLE25* (*VIT_01s0026g01090*), *BAM1* (*VIT_00s1353g00010*) and *BAM3* (*VIT_01s0010g00330*) were not induced by WD in roots or shoots of any *Vitis* genotype in our study (data not shown). In Arabidopsis, the expression induction of *CLE25* is observed after 3 h of dehydration [57]. This rapid molecular response could have already taken place in the roots of grapes harvested after 7 and 14 days of WD stress and was then not detected anymore at the transcriptomic level.

Other recent findings showed that WD increases sulfate concentrations in the xylem [28, 58, 59], which then induces an increase in cysteine biosynthesis resulting in increased *NCED3* expression, increased ABA biosynthesis and stomatal closure [28]. Our study indicated that cysteine biosynthesis may have been increased in the roots of RM and this may have contributed to the increased *NCED3* transcript abundance in response to WD.

Root-to-shoot long-distance signaling is a complex communication system involved in several crucial processes for plant growth [60]. There is evidence in grapevine that root signaling occurs before observed changes in transcript abundance in response to a gradual WD; proteomic analysis revealed an increase in protein abundance associated with photosynthesis and antioxidant defenses in the shoot tip (including immature leaves) prior to declines in shoot elongation rate, stomatal conductance and photosynthesis [61].

In this study, a stronger response was observed in the leaves involving many DEGs related to stress and ABA as observed previously [12, 32]. A set of DEGs was detected showing a strong increase in expression in both roots and leaves by WD and a significantly elevated transcript abundance in RM leaves relative to the three other genotypes. Interestingly, many genes related to ABA and involved in the common core response to WD shared by the four genotypes presented in Fig. 6 were included in this list of genes. Several other genes known to be regulated in response to drought were included (Additional file 18). Numerous genes, including the hub genes, highly correlated to the modules darkturquoise and royalblue, WGCNA gene clusters significantly associated to the WD treatment in the roots (Fig. 8), were present in this gene set, such as *PP2C4* (*ABI1*), hub gene of the royalblue module (Fig. 11b, Additional files 13 and 18). Also, it is interesting that specific genes encoding MIPs in RM leaves after two weeks of WD treatment showed a strong downregulation in this condition (Fig. 7). Among these genes, *PIP2–7* showed a strong downregulation only in RM. In parallel, the gene *TSPO* was found to be specifically strongly induced in the same condition (Fig. 11e). This negative association is known at the protein level with a reduction of PIP2–7 abundance through an AQPs regulatory mechanism involving TSPO [62]. Altogether, these observations indicated that the perception or the sensitivity of RM was more pronounced than in the other *Vitis* genotypes. The root transcriptome modification of this genotype was tightly linked to strong transcriptional regulation in the shoot, which may contribute to its higher drought tolerance.

## Conclusion

WD experiments in this study were consistent with previous studies indicating that RM was more drought tolerant than the other three *Vitis* genotypes. The superior drought tolerance of RM to the other genotypes was associated with higher stomatal conductance and photosynthesis at equivalent WD. WGCNA identified gene subnetworks associated with the responses to WD including ABA biosynthesis and signaling subnetworks, lipid metabolism, antioxidant defense, AQPs, and heat shock proteins. Drought tolerance in RM was correlated with higher sensitivity to WD with an early and enhanced expression of ABA biosynthesis and signaling genes. Decreased AQP expression in WD RM leaves may also contribute to its drought tolerance. Higher tolerance to WD also included higher photosynthesis for RM, which may contribute to long-term energy supplies. Improved photosynthesis in WD RM may be associated with a putative higher stem water potential and ABA-increased expression of *GOLS2* allowing for improved antioxidant defense and subsequent improved photochemical efficiency in the leaves.

## Methods

### Plant material and growth conditions

For each experiment, four genotypes were studied; *Vitis vinifera* cv. Cabernet Sauvignon clone 8 (CS), *Vitis riparia* (RG), *Vitis champinii* (RM) and a *Vitis vinifera* x *girdiana* hybrid (SC). The original vegetatively-propagated vines were provided by Dr. Andrew Walker and the Foundation Plant Services at UC Davis and continually repropagated in our own glass greenhouse. Vines were grown in the greenhouse at 21–26 °C, 20–50% relative humidity and average mid-day light intensities of 1200 μmoles m^− 2^ s^− 1^. Supplemental light was applied using 1000 W high pressure sodium lamps to maintain a 16 h light and 8 h dark cycle. Vines of each genotype were selected and pruned for similar sizes among 1 to 3-year-old vines grown in 12 L pots containing equal soil mass from the bottom of the pot to the top of the pot with 0.5 kg clay balls, 0.5 kg fritted clay and 13.3 kg medium sand. They were irrigated with Cramer’s full nutrient solution (1.5 mM Ca (NO_3_)_2_, 4H_2_O; 2 mM KNO_3_; 0.6 mM Mg (SO_4_), 7H_2_O; 1 mM KH_2_PO_4_; 1.5 mM CaCl_2_, 2H_2_O; 36 μM Fe (Sprint 330); 1 μM MnSO_4_, H_2_O; 0.5 μM CuSO_4_, 5H_2_O; 20 μM ZnSO_4_, 7H_2_O; 20 μM H_3_BO_3_; 0.01 μM (NH_4_)_6_Mo_7_O_24_, 4H_2_O) twice a week before the experiment began. A shoot of each vine was pruned to form a single upright shoot tied to a stake. The apical shoot tip was removed and the shoot was left with 10 leaves remaining. Lateral buds were removed except for one axillary stem growing at the 10th node. Any other lateral branches that emerged were removed. This allowed for relatively similar leaf area for each vine to enable uniform control of transpiration and RSWC during the WD experiments. In other experiments utilizing smaller vines and pots, one-node cuttings of the four different genotypes were rooted in perlite/vermiculite mix 1/1. Rooted cuttings were transferred into 0.96 L pots containing 80 g of fritted clay and completed with 1 kg of medium sand. They were watered for at least four weeks with a full nutrient solution before the start of the experiment. Plants used for RNA-Seq were irrigated with Gibeaut’s solution [63].

### WD experiments, physiological measurements and sample collection

Prior to each experiment, pots were weighed and then watered to reach 100% relative soil water content at field capacity (RSWC). One hundred percent RSWC was defined as the weight of the individual pot two hours after a saturating irrigation when excess water was removed by gravity. Each pot was covered with aluminum foil to minimize soil evaporation. Using plants aged between one and three years old, one experiment consisted of a set of vines which was kept at 100% RSWC for 13 days as the control by weighing each pot daily and adding nutrient solution to replace the water lost. Another set of vines were exposed to a water deficit treatment by allowing the pots to dry down naturally and then re-watering them on the 8th day to reach 100% RSWC. For the second experiment, a set of these aged vines was kept at 100% RSWC for three weeks as a control, while another plant set was dried down naturally until it reached 50% RSWC and then maintained at 50% RSWC for the water deficit treatment. Pots were weighed daily. Every 2nd day, stem lengths were measured. Photosynthesis and stomatal conductance were determined using a portable photosynthesis system (LiCOR model 6400XT, Lincoln, NE, USA) set at 400 μmol s^− 1^ flow rate, 400 μmol mol^− 1^ reference CO_2_, leaf at 27 °C and PAR 1000 μmol m^− 2^ s^− 1^. Stem water potential was measured with a Plant Water Status Console (Soilmoisture Equipment Corp., CA, USA) pressure chamber using excised leaves previously covered in a plastic zip locked bag covered with aluminum foil for 2 h before measurement. At the end of this experiment, root hydraulic conductivity measurements of the vines were obtained as described previously [64]. Briefly, root systems of the four genotypes were studied at the same time using a four-chamber extension (PSM Instrument Company, Albany, OR, USA). Root hydraulic conductivity was calculated using pressure-flow relationships with exudates collected using a gauze connected to the stem (after at least 2 min to obtain a steady-state flow) with pressure increments of 0.05 MPa until a final pressure of 0.25 MPa. RNA-Seq samples were collected from a separate experiment performed on young cuttings of each genotype. Vines were divided into two plant sets, one maintained at 100% RSWC and another dried down naturally for two weeks. Leaf blades (without petiole) and the entire root system were collected from both treatments represented by three to five individual vines as experimental replicates for each genotype at day 7 and day 14 (except for RG roots in control conditions after one week of treatment, represented by two experimental replicates only due to sample loss and RNA-Seq pre-processing analysis). Samples were immediately frozen in liquid nitrogen and stored at − 80 °C.

### RNA extraction, library preparation and sequencing

Total RNA was extracted from ground samples using a cetyltrimethylammonium bromide (CTAB)- based method [65] and purified with the Spectrum plant total RNA kit (Sigma) according to the manufacturer’s instructions. Total RNA was treated with RNAse-free DNAse I (Qiagen) and quantified by A260 absorbance using a Nanodrop spectrophotometer (Thermo Scientific NanoDrop 2000a). Aliquots of the RNA samples were sent to the Nevada Genomics Center, the RNA quality was assessed with the Plant RNA Nano assay on an Agilent 2100 Bioanalyzer and quantity was determined more precisely using a Ribogreen quantification system. The samples were sent to the Oklahoma Medical Research Foundation Genomics center for sequencing using Illumina NextSeq technology.

### Pre-processing of RNA-Seq data

Illumina adapters were removed from the single end 75 bp raw reads using Trimmomatic v0.36 [66]. The reads were quality-trimmed using a minimum Phred quality score of 5, at the leading end, trailing end, and along a four-base sliding window, with a minimum trimmed length of 36 bp. Library quality of each sample was assessed with FastQC v0.11.5 before and after trimming [67]. Filtered reads were aligned against the V1 grapevine genome [37] using HISAT2 v2.0.5 [68] with the “downstream-transcriptome-analysis” output setting. Counts per gene were obtained with featureCounts v1.5.1 of the subread toolset [69], with uniquely aligned reads summarized once per annotated gene. Reads aligned to multiple genomic locations and reads mapped to locations with more than a single gene feature were not counted.

### Differential expression analysis and functional categories enrichment

The R package DESeq2 [70] was used to identify differentially expressed genes (DEGs) using the following threshold: False Discovery Rate (FDR) adjusted p-value < 0.05. To select genes strongly induced by the WD, DEGs with a log fold change (LFC) > 4 in both leaves and roots were selected. From these sets, expressed genes were filtered to omit genes with an average TPM below 20 across all samples. Comparisons were then performed between genotypes to detect and extract genes that were significantly induced in a given genotype. Gene Ontology (GO) enrichment was performed using the R package topGO [71]. Enriched functional categories with an FDR adjusted p-value > 0.01 after the Fisher’s test were filtered for further analysis. BIN codes were attributed to each gene using Mercator 4 (http://plabipd.de/portal/mercator-ii-alpha-version-, last accessed 09/29/17) and the enrichment analysis was performed using Fisher’s test. Enriched functional categories with a Bonferroni’s adjusted p-value > 0.05 and an enrichment ≤1 were filtered for further analysis. Expression heatmaps were drawn using the R packages ComplexHeatmap v1.18.0 [72], dendextend [73], circlize [74]. For clustering analysis on heatmaps, distances were computed by the z-scores using the R function dist with the “Euclidean” distance method. A hierarchical clustering was performed on these distances with the R function hclust using the “complete” method. UpSet plots were produced using the R package UpSetR [75]. All the other plots were generated using the R package ggplot2 [76].

### Co-expression network analysis

A gene co-expression network was constructed using the WGCNA R package (v1.63) [35, 77] using all the libraries for each organ, leaves (60) and roots (57). Prior to this analysis, low-expressed genes were removed with a minimum threshold of 20 counts in all the libraries. A total of 21,216 and 21,542 genes satisfying the above threshold were obtained for the roots and the leaves, respectively. Count data were transformed using the function varianceStabilizingTransformation of the R package DESeq2. The resulting set of counts was used for network construction and module detection using the function blockwiseModules. Briefly, an adjacency matrix was created by calculating the biweight mid-correlation raised to a power β of 8 for both organs (soft threshold estimated with the pickSoftThreshold function to ensure to fit a scale free topology network) and the maxPoutliers parameter set to 0.05. The subsequent Topological Overlap Matrix (TOM) was used for module detection using the DynamicTreecut algorithm with a minimal module size of 30 and a branch merge cut height of 0.25. The module eigengenes were used to evaluate the association among the resulting modules (58 for the roots and 44 for the leaves) and traits (Genotype, Treatment, Harvesting time).

### RT-qPCR

Total RNA was reverse transcribed into cDNA using the iScript reverse transcription supermix for RT-qPCR (Bio-Rad). Quantitative PCR reactions were performed using iTaq universal SYBR green supermix on a CFX96 Touch Real-Time PCR detection system (Bio-Rad) according to the manufacturer recommendations. Normalized relative quantities were determined according to Hellemans et al. [78] using the reference genes *GAPDH* and *ACT7*. Relative quantities are expression values relative to the average expression for a given gene. Normalized relative quantities (NRQs) are relative quantities per sample relative to the geometric mean of the reference genes. The means were calculated averaging the biological sample NRQs. Primer sequences are listed in Additional file 19.

### Statistical analysis

All statistical analyses were performed using the R software [79]. For comparisons between two means, the Wilcoxon non-parametric test was used. For comparisons among more than two means, a 2-way ANOVA was performed. When the assumptions for a parametric test were not respected (e.g. non-normal), a multiple comparison test was performed after a non-parametric Kruskal-Wallis test using the function kruskalmc from the pgirmess R package [80]. Letters to indicate significant differences among multiple comparisons were obtained using the function multcompLetters from the multcompView R package [81].

## Additional files

Additional file 1: Stem water potential of the four *Vitis* species during the recovery experiment. (PDF 63 kb)

Additional file 2: Statistics for the WD recovery experiment at day 6 and 8. (XLSX 13 kb)

Additional file 3: Statistics for the long-term WD experiment from day 12 to day 20. (XLSX 22 kb)

Additional file 4: Water relations measurements of the four *Vitis* species with long-term WD maintained at 50% RSWC. (PDF 77 kb)

Additional file 5: Physiological measurements on small potted propagated vines of the four *Vitis* genotypes in response to severe WD. (PDF 127 kb)

Additional file 6: Differential expression analysis results per genotype, organ and week. (XLSX 27292 kb)

Additional file 7: Functional categories representative of a response to WD. (XLSX 10 kb)

Additional file 8: Gene Ontology enrichment analysis of Biological Process category on Venn gene lists. (XLSX 307 kb)

Additional file 9: BIN code enrichment analysis on Venn gene lists. (XLSX 99 kb)

Additional file 10: DEA and TPM values for the ABA related genes from comparison results between drought treated and control vines for each organ x week x genotype combination. (XLSX 97 kb)

Additional file 11: Normalized relative quantities (RT-qPCR) for *NCED3* and *DHN1* in young propagated vines in response to WD. (PDF 79 kb)

Additional file 12: DEA and TPM values for the MIP family genes from comparison results between drought treated and control vines for each organ x week x genotype combinations. (XLSX 62 kb)

Additional file 13: Gene module membership in the root samples. (XLSX 45939 kb)

Additional file 14: Summary of the WGCNA modules in the roots. (XLSX 36 kb)

Additional file 15: Expression profile of the gene *SAT3* highly connected to the root WGCNA module skyblue3. (PDF 69 kb)

Additional file 16: Gene module membership in the leaf samples. (XLSX 34506 kb)

Additional file 17: Summary of the WGCNA modules in the leaves. (XLSX 29 kb)

Additional file 18: Genes strongly induced by the WD in the roots and the shoot of RM. (XLSX 30 kb)

Additional file 19: Primer list. (XLSX 9 kb)

## Data Availability

RNA-Seq data were deposited in the Sequence Read Archive (SRA) database with the accession number PRJNA516950.

## Abbreviations

AAE3: ACYL-ACTIVATING ENZYME 3
ABA: Abscisic acid
ABA-GE: ABA-glucose ester
ABCG: ATP binding cassette G
ABF: ABRE-BINDING FACTOR
ABI: ABA INSENSISTIVE
ADS3: FATTY ACID DESATURASE 5
AP2-ERF: APETALA2-ETHYLENE RESPONSIVE FACTOR
AQP: Aquaporin
ATAF1: ARABIDOSIS THALIANA ACTIVATING FACTOR 1
BAM: BARELY ANY MERISTEM
BIN: BIN code
CASPL 1D1: CASP-LIKE PROTEIN 1D1
CLE: CLAVATA3/EMBRYO-SURROUNDING REGION-RELATED
CS: Cabernet Sauvignon
CTAB: Cetyltrimethylammonium bromide
CYP: CYTOCHROME P450
DEG: Differentially expressed gene
DHN1: DEHYDRIN 1
DREB: DEHYDRATION RESPONSE ELEMENT-BINDING
FDR: False discovery rate
GDSL: GDSL-MOTIF ESTERASE/ACYLTRANSFERASE/LIPASE
GEA6: EARLY METHIONINE-LABELLED 6 PROTEIN
GO: Gene ontology
GOLS: GALACTINOL SYNTHASE
GPAT: GLYCEROL-3-PHOSPHATE ACYLTRANSFERASE
HAI2: HIGHLY ABA-INDUCED PP2C GENE 2
HSP: Heat shock protein
KCR: BETA-KETOACYL REDUCTASE
KCS: 3-KETOACYL-COA-SYNTHASE
LEA: LATE EMBRYOGENESIS ABUNDANT
LFC: Log fold change
MIP: Major intrinsic protein
NAC: NAC DOMAIN CONTAINING
NCED: Nine-cis-epoxycarotenoid dioxygenase
NPF: NITRATE TRANSPORTER 1/PEPTIDE TRANSPORTER FAMILY
PCA: Principal component analysis
PIP: PLASMA MEMBRANE INTRINSIC PROTEIN
PP2C: Protein phosphatase 2C
RAB: RAB GTPASE
RCAR: REGULATORY COMPONENTS OF ABA RECEPTOR
RD: RESPONSIVE TO DESICCATION
RG: Riparia Gloire
RM: Ramsey
RNA-Seq: RNA sequencing
RSWC: Relative soil water content
RT-qPCR: Reverse transcription quantitative polymerase chain reaction
SAT: SERINE ACETYLTRANSFERASE
SC: SC2
SER: Shoot elongation rate
SNRK: SNF1-RELATED PROTEIN KINASE
SQD: SULFOQUINOVOSYLDIACYLGLYCEROL
TF: Transcription factor
TIP: TONOPLAST INTRINSIC PROTEIN
TOM: Topological overlap matrix
TPM: Transcript per million
TSPO: TSPO RELATED PROTEIN
VAS: LIPID TRANSFER-LIKE PROTEIN VAS
WD: Water deficit
WGCNA: Weighted gene co-expression network analysis
XERO: DEHYDRIN XERO
XIP: X INTRINSIC PROTEIN
