# Supplementary material for: Drought tolerance of the grapevine, Vitis champinii cv. Ramsey, is associated with higher photosynthesis and greater transcriptomic responsiveness of abscisic acid biosynthesis and signaling
Source: BMC Plant Biol. 2020 Feb 4;20:55. doi: 10.1186/s12870-019-2012-7 (PMC7001288; doi:10.1186/s12870-019-2012-7)
Supplement: Supplementary file 1 — Stem water potential of the four Vitis species during the recovery experiment. (PDF 63 kb) [file 12870_2019_2012_MOESM1_ESM.pdf]

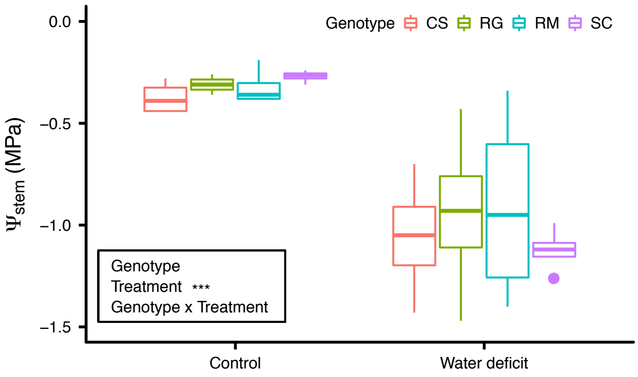

Additional file 1: Stem water potential of the four *Vitis* species during the recovery experiment.

Stem water potential measured after six days of treatment on old vines of the four genotypes during the recovery experiment. For each condition (i.e. genotype x treatment), data are represented using boxplots, n = four individual plants. ***p-value<0.001; 2-way ANOVA. Red, green, blue and purple colors correspond to CS, RG, RM and SC, respectively.
