# Supplementary material for: Drought tolerance of the grapevine, Vitis champinii cv. Ramsey, is associated with higher photosynthesis and greater transcriptomic responsiveness of abscisic acid biosynthesis and signaling
Source: BMC Plant Biol. 2020 Feb 4;20:55. doi: 10.1186/s12870-019-2012-7 (PMC7001288; doi:10.1186/s12870-019-2012-7)
Supplement: Supplementary file 4 — Water relations measurements of the four Vitis species with long-term WD maintained at 50% RSWC. (PDF 77 kb) [file 12870_2019_2012_MOESM4_ESM.pdf]

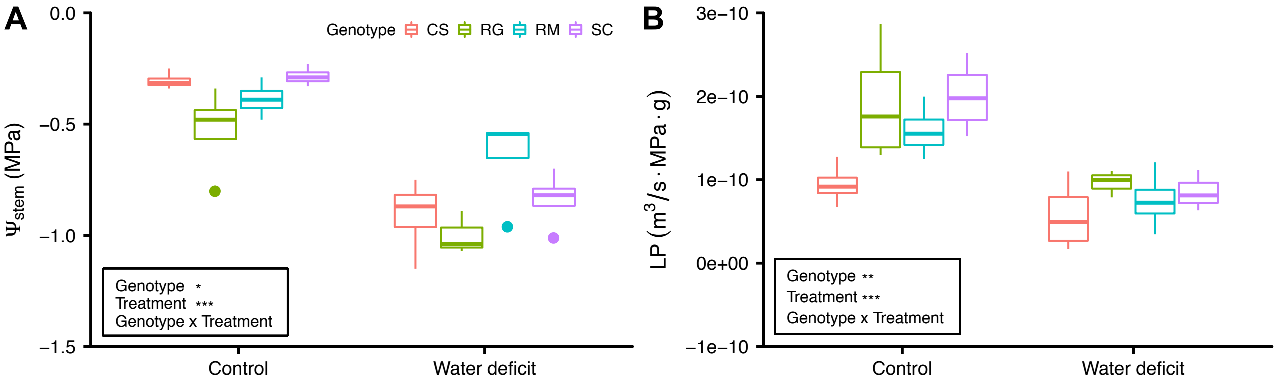

Additional file 4: Water relations measurements of the four *Vitis* species with long-term WD maintained at 50% RSWC.

A) Stem water potential measured after 18 days of treatment on old vines of the four genotypes. B) Root hydraulic conductivity measured at day 20. For each condition (i.e. genotype x treatment), data are represented using boxplots, n = three-four individual plants. *p-value<0.05, **p-value<0.01, ***p-value<0.001; 2-way ANOVA. Red, green, blue and purple colors correspond to CS, RG, RM and SC, respectively.
