# Supplementary material for: Drought tolerance of the grapevine, Vitis champinii cv. Ramsey, is associated with higher photosynthesis and greater transcriptomic responsiveness of abscisic acid biosynthesis and signaling
Source: BMC Plant Biol. 2020 Feb 4;20:55. doi: 10.1186/s12870-019-2012-7 (PMC7001288; doi:10.1186/s12870-019-2012-7)
Supplement: Supplementary file 5 — Physiological measurements on small potted propagated vines of the four Vitis genotypes in response to severe WD. (PDF 127 kb) [file 12870_2019_2012_MOESM5_ESM.pdf]

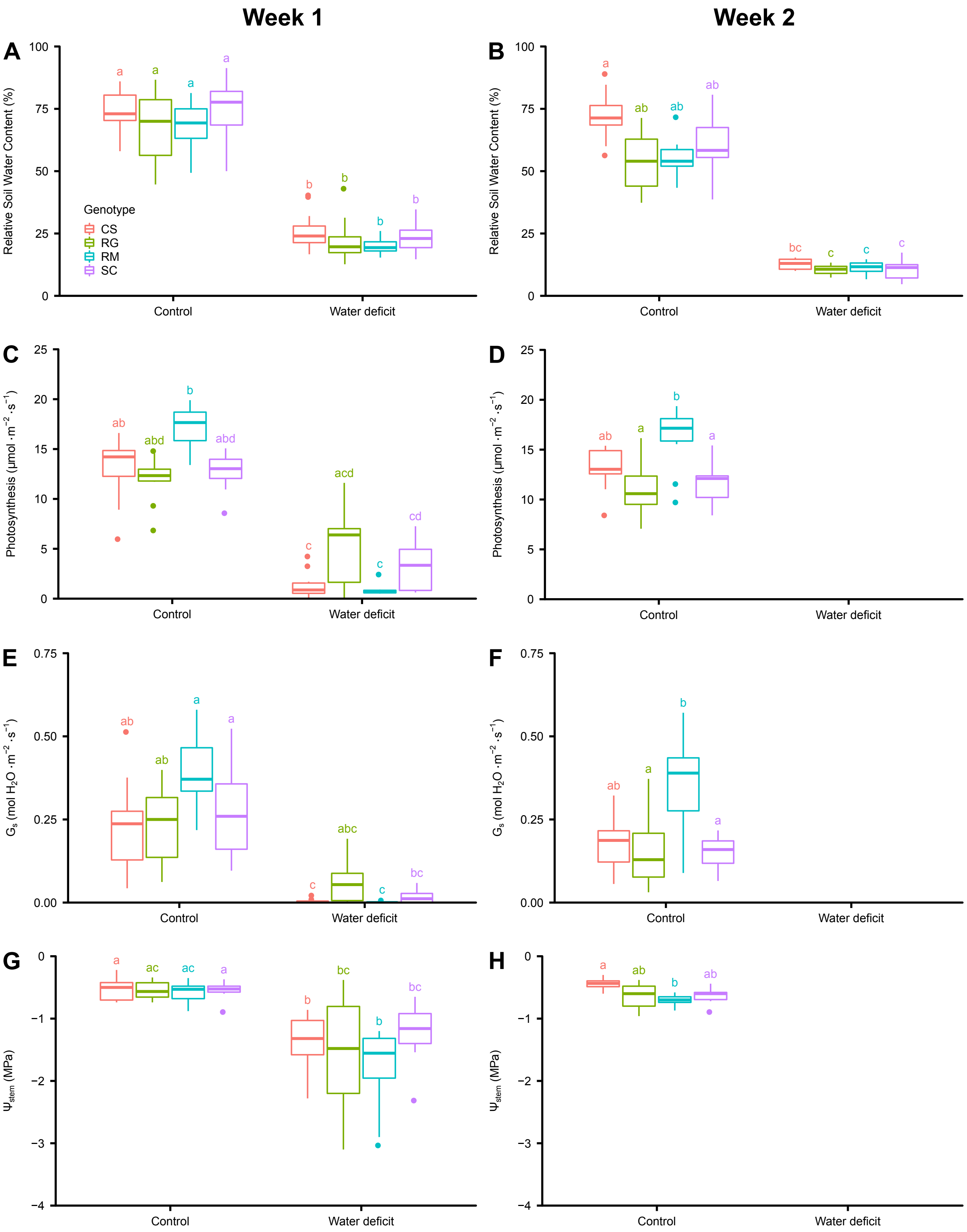


**Additional file 5: Physiological measurements on small potted propagated vines of the four *Vitis* genotypes in response to severe WD.**

For the RSWC, each condition (i.e. genotype x treatment) is represented by twenty individual plants in Week 1 and ten plants for the Week 2. For the other variables, each condition is represented by ten plants. Letters indicate significant differences between conditions using a multiple comparison test after Kruskal-Wallis (p-value<0.05). Red, green, blue and purple colors correspond to CS, RG, RM and SC, respectively. WD-treated vines died in the Week 2, making it impossible for some physiological measurements.
