# Supplementary material for: Drought tolerance of the grapevine, Vitis champinii cv. Ramsey, is associated with higher photosynthesis and greater transcriptomic responsiveness of abscisic acid biosynthesis and signaling
Source: BMC Plant Biol. 2020 Feb 4;20:55. doi: 10.1186/s12870-019-2012-7 (PMC7001288; doi:10.1186/s12870-019-2012-7)
Supplement: Supplementary file 11 — Normalized relative quantities (RT-qPCR) for NCED3 and DHN1 in young propagated vines in response to WD. (PDF 79 kb) [file 12870_2019_2012_MOESM11_ESM.pdf]

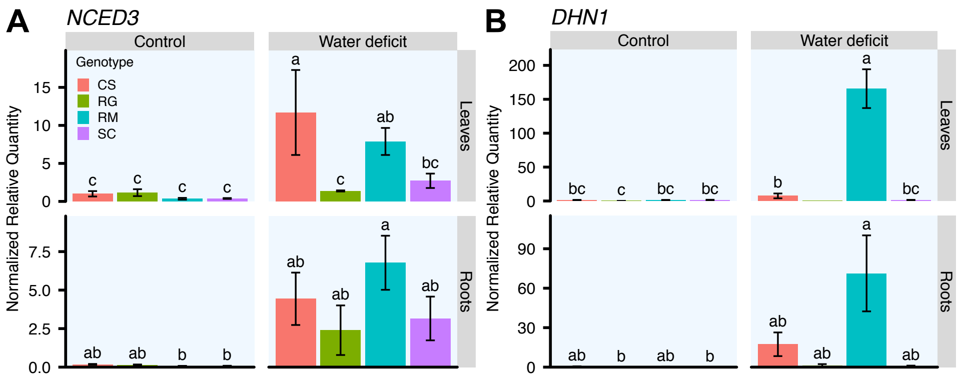

Additional file 11: Normalized relative quantities (RT-qPCR) for *NCED3* and *DHN1* in young propagated vines in response to WD.

Expression profiles of *NCED3* (A) and *DHN1* (B) after one week of WD treatment in young propagated vines. Expression in the control (left column) and WD treated vines (right column) for the leaves (first row) and the roots (second row) is represented as a normalized relative quantity, mean ± SE, n = three-four individual vines (except for RG with two individual plants for the WD condition in the leaves). Letters indicate significant differences between conditions using a Tukey's HSD test after ANOVA or a multiple comparison test after Kruskal-Wallis (p-value<0.05). Red, green, blue and purple colors correspond to CS, RG, RM and SC, respectively.
