# Supplementary material for: Drought tolerance of the grapevine, Vitis champinii cv. Ramsey, is associated with higher photosynthesis and greater transcriptomic responsiveness of abscisic acid biosynthesis and signaling
Source: BMC Plant Biol. 2020 Feb 4;20:55. doi: 10.1186/s12870-019-2012-7 (PMC7001288; doi:10.1186/s12870-019-2012-7)
Supplement: Supplementary file 15 — Expression profile of the gene SAT3 highly connected to the root WGCNA module skyblue3. (PDF 69 kb) [file 12870_2019_2012_MOESM15_ESM.pdf]

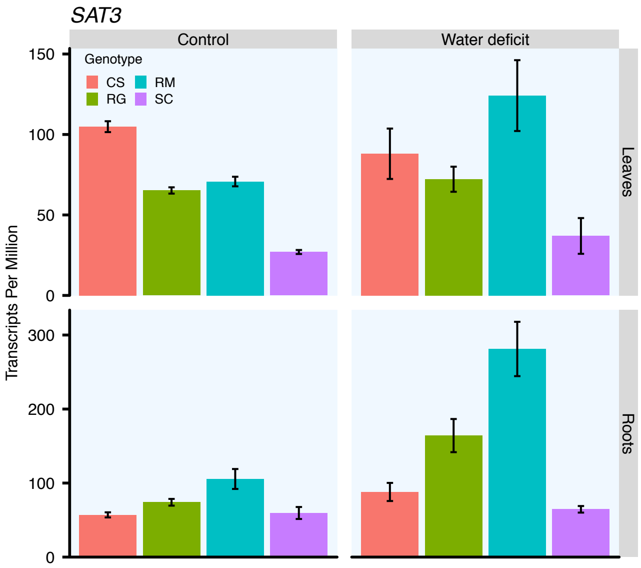

Additional file 15: Expression profile of the gene *SAT3* highly connected to the root WGCNA module skyblue3.

Expression of *SAT3* after two weeks of treatment. Expression in control (left column) and WD treated vines (right column) for the leaves (top row) and the roots (bottom row) is represent in transcripts per million, mean ± SE, n = three-five individual vines. CS, RG, RM and SC are represented in red, green, blue and purple respectively.
